# Neurobiology of schizophrenia: from outcome to pathophysiological insights

**DOI:** 10.1007/s00406-012-0294-y

**Published:** 2012-01-24

**Authors:** P. Falkai, H. -J. Möller

**Affiliations:** 1 Department of Psychiatry and Psychotherapy, University of Göttingen, Von-Siebold-Str. 5, 37075 Göttingen, Germany; 2 Department of Psychiatry, Ludwigs-Maximililans-University Munich, Nussbaumstr. 7, 80336 Munich, Germany

Besides positive and negative symptoms, the schizophrenia syndrome is characterized by various deficits in cognitive domains. This progressive neurocognitive impairment deeply impacts the lives of those afflicted and their families and is responsible for worsening of social abilities. In a large-scale naturalistic open-label study of first-episode schizophrenia patients, Segarra et al. [1] have proven that men have an earlier age of onset, poorer premorbid functioning and a higher presence of prodromal and negative symptoms compared with women. Women again show a better social outcome with more stabile relationships. Especially among women, acute stress precedes the onset of psychosis. However, at the 2-year follow-up under risperidone treatment, in both genders, the outcome improved significantly, which was more pronounced in men. However, in this study, non-deficit and chronic schizophrenia patients have been investigated. Réthelyi et al. [2] investigated cognitive functioning including working memory, attention, short-term memory, verbal memory and cognitive flexibility in these both groups and found that patients with deficit schizophrenia suffer from more severe neurocognitive impairment with cognitive flexibility being an independent predictor of outcome. The genetic background may influence cognitive deficits in these patients. Accordingly, Lennertz et al. [3] show the T allele of a promoter variant of the SHANK1 gene leading to impairments in working memory in schizophrenia patients and subjects at risk for psychosis. This is an interesting finding considering that SHANK1 knockout mice entail altered memory functions along with reduced dendritic spines and postsynaptic density, which also are features of schizophrenia. Cognitive test performance also serves as separator between schizophrenia and bipolar disorder. In this context, inhibitory control, which includes aspects of cognitive and behavioral control in processes of maintaining contextual information, may discriminate schizophrenia from affective disorders. Christodoulou et al. [4] detected more pronounced deficits of inhibitory control in schizophrenia patients with familial risk compared to patients with bipolar disorder.

One less familiar feature of schizophrenia is the occurrence of motor deficits. Spontaneous parkinsonism including bradykinesia, rigidity and tremor was investigated by Peralta et al. [5] in 200 drug-naïve first-episode schizophrenia patients. The most frequent sign was rigidity, followed by bradykinesia, which was related to negative symptoms. Rigidity was related to neurological soft signs and tremor to dyskinetic movements. The authors found the NINDS criteria for spontaneous parkinsonism being the most suitable instrument to assess these symptoms. Second-generation antipsychotics induce less motor symptoms, but extrapyramidal adverse events and other side effects such as weight gain and prolactin-related symptoms have been found to be more frequently present in patients treated with risperidone long-lasting injectable (RLAI) compared to aripiprazole treatment by de Arce Cordόn et al. [6]. However, relapse occurred in 27% of the patients treated with aripiprazole and only 16% of the RLAI-treated patients. Riedel et al. [7] highlight ethical and medico-legal aspects of clinical trials of new drugs with special focus on placebo-controlled trials and withdrawal conditions in schizophrenia patients. The Trial Criteria in Schizophrenia Working Group defined consensus criteria that are important to ensure the patient’s safety. These criteria should be evaluated using standardized rating scales applying established cutoff criteria. Another aspect of burden of disease is covered by the study of Möller-Leimkühler and Wiesheu [8]. They investigated caregivers, mainly mothers of adult children suffering from schizophrenia, and found expressed emotions and perceived social support predictive for caregiver’s burden, whereas, with regard to the patient’s variables, regular employment reduced caregiver’s distress. The authors conclude that family interventions should improve dysfunctional interactions and enhance the carers’ social activities.

Despite enormous efforts in neuroscience research, the pathophysiology of schizophrenia is not well understood. Varadarajulu et al. [9] investigated expression of the histidine triad nucleotide-binding protein-1 (HINT1) in the dorsolateral prefrontal cortex and thalamus of schizophrenia patients. HINT1 was downregulated in the prefrontal cortex and upregulated in the thalamus. Since this protein is important in control of transcriptional processes, cellular stress and apoptosis, it may represent a potential target for future treatment studies in animal models. In the pathophysiology of schizophrenia, an inflammatory process since some decades stays in focus. Aslan et al. [10] start an alternative approach by investigating IgG antibodies to the West Nile virus in 112 schizophrenia patients and 162 controls in Turkey. These antibodies were detected in 6 schizophrenia patients and 5 controls, suggesting no relationship between this virus and schizophrenia symptoms. Albeit, further prospective studies investigating the relationship between environmental factors like virus diseases and obstetric complications during neurodevelopment and onset of disease during young adulthood are needed to elucidate pathophysiological aspects of psychosis.
